# Polarization of Tumor Cells and Tumor‐Associated Macrophages: Molecular Mechanisms and Therapeutic Targets

**DOI:** 10.1002/mco2.70372

**Published:** 2025-09-01

**Authors:** Guohao Wei, Bin Li, Mengyang Huang, Mengyao Lv, Zihui Liang, Chuandong Zhu, Lilin Ge, Jing Chen

**Affiliations:** ^1^ Department of Oncology The Second Hospital of Nanjing Nanjing University of Chinese Medicine Nanjing China; ^2^ The Comprehensive Cancer Center, Department of Central Laboratory, The Affiliated Huai'an No.1 People's Hospital Nanjing Medical University Huai'an China; ^3^ Department of Biochemistry and Molecular Biology, School of Medicine, Nanjing University of Chinese Medicine, No.138 Xianlin Avenue Nanjing University of Chinese Medicine Nanjing China; ^4^ Jiangsu Province Engineering Research Center of Traditional Chinese Medicine Health Preservation Nanjing University of Chinese Medicine Nanjing China

**Keywords:** metabolism reprogramming, TAMs, TME, tumor cell

## Abstract

Tumor‐associated macrophages (TAMs) are prominent constituents of solid tumors, and their prevalence is often associated with poor clinical outcomes. These highly adaptable immune cells undergo dynamic functional changes within the immunosuppressive tumor microenvironment (TME), engaging in reciprocal interactions with malignant cells. This bidirectional communication facilitates concurrent phenotypic transformation: tumor cells shift toward invasive mesenchymal states, whereas TAMs develop immunosuppressive, pro‐tumorigenic traits. Increasing evidence highlights metabolic reprogramming, characterized by dysregulation of lipid metabolism, amino acid utilization, and glycolytic activity, as the fundamental molecular basis orchestrating this pathological symbiosis. However, a comprehensive understanding of how metabolic reprogramming specifically coordinates the mutual polarization of tumor cells and TAMs is lacking. This review thoroughly examines the molecular mechanisms governing this co‐polarization process, detailing critical transcriptional regulators, essential signaling pathways, and the maintenance of adaptive phenotypes within the TME. Furthermore, this review critically assesses promising therapeutic strategies aimed at disrupting this alliance, including the use of metabolically targeted agents, engineered chimeric antigen receptor macrophages, and TAM‐selective nanoparticle delivery systems. These insights provide a crucial foundation for the development of next‐generation cancer immunotherapies focused on reprogramming pathological polarization dynamics to overcome treatment resistance and improve clinical outcomes.

## Graphical abstract

Bidirectional metabolic interactions between tumor cells and TAMs in the TME metabolic reprogramming facilitates reciprocal crosstalk between TAMs and tumor cells within the immunosuppressive TME. This interaction promotes both the pro‐tumor functions of TAMs and tumor mesenchymal transition through complex molecular signaling.

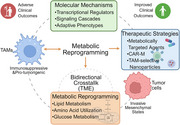

## 1 Introduction

Cancer remains a leading cause of death worldwide, posing a significant challenge to global health [1]. The tumor microenvironment (TME) is now recognized as an active, co‐evolving ecosystem essential for cancer progression and therapy resistance [2, 3, 4]. Within this setting, cellular plasticity drives tumor evolution. This plasticity is prominently exhibited by both malignant cells undergoing processes such as epithelial‒mesenchymal transition (EMT) and key immune constituents, most notably tumor‐associated macrophages (TAMs), which are defined as macrophages residing within or recruited to the tumor mass [5, 6, 7, 8]. TAMs exhibit characteristic surface markers (e.g., elevated CD206 and CD163) and functional programs distinct from those of their tissue‐resident or circulating counterparts, enabling them to profoundly influence the TME landscape [7, 9, 10, 11].

The pivotal role of macrophages in cancer was first suggested by Rudolf Virchow in the 19th century, who noted the frequent presence of leukocytes in tumors. However, the modern conceptualization of TAMs as active participants in tumor biology emerged in the late 20th and early 21st centuries. Seminal studies have established their functional dichotomy: while they are capable of anti‐tumor activity (M1‐like), TAMs in established tumors predominantly adopt a spectrum of immunosuppressive and pro‐tumorigenic states (M2‐like), promoting angiogenesis, tissue remodeling, metastasis, and therapy resistance [7, 12]. This shift is a dynamic adaptation driven by complex interactions with tumor cells and other TME components [10, 13]. Metabolic reprogramming is central to this crosstalk and TME function [14, 15, 16]. Tumor cells extensively alter their metabolism, including dysregulating lipid metabolism, to fuel proliferation and survival [17, 18, 19]. This creates nutrient competition, acidifies the microenvironment via lactate accumulation, and profoundly impacts immune cell metabolism and function, especially in TAMs [20, 21]. In addition to serving as energy sources, lipids act as potent signaling molecules. They directly modulate TAM polarization and immunosuppressive function through receptors such as PPARγ and Toll‐like receptor 4 (TLR4) [22, 23]. Recent evidence highlights a sophisticated “metabolic symbiosis” within the TME characterized by reciprocal lipid exchange and metabolic reprogramming between tumor cells and TAMs. Tumor cells influence TAM lipid uptake and metabolism, leading to lipid droplet accumulation. Conversely, TAMs process and supply specific lipids that increase tumor cell invasiveness and metastasis, positioning them as central metabolic hubs [24, 25, 26, 27].

Despite advances in TAM biology and metabolism, systematic analyses specifically focusing on how metabolic reprogramming orchestrates the reciprocal polarization of tumor cells and TAMs to drive progression and therapeutic resistance remain limited. Foundational knowledge often stems from studies on in vitro polarized macrophages or isolated pathways, leaving gaps in understanding integrated, dynamic crosstalk within the complex human TME [28, 29]. This review synthesizes the current understanding of this axis. We propose that targeting molecular switches that govern this co‐adaptive plasticity, particularly lipid‐centered metabolic symbiosis, represents a promising strategy that is potentially more effective than attacking fixed cell states.

This review will initially delineate the defining characteristics and polarization states of TAMs, briefly revisiting significant historical milestones in their discovery and functional characterization. We will subsequently elucidate the principal molecular mechanisms underlying the adaptive phenotypes of both tumor cells and TAMs. These mechanisms involve key transcriptional regulators, essential signaling pathways such as phosphoinositide 3‐kinase (PI3K)/AKT and CSF‐1R, and significant metabolic reprogramming involving glycolysis, fatty acid oxidation (FAO), and lipid synthesis. A central focus will be the mediated crosstalk that sustains their mutual reinforcement within the TME. Finally, we will examine emerging therapeutic strategies aimed at disrupting this polarization axis. These strategies include metabolic interference targeting molecules such as CD36 or inhibition of FAO, as well as novel approaches such as engineered macrophages, highlighting their potential to overcome therapy resistance and improve cancer outcomes.

## 2 Dynamic Tumor Microenvironment: Beyond Genetic Mutations

The TME functions as a dynamic ecosystem in which immune cells, stromal components, the ECM, the vasculature, and signaling molecules collectively drive cancer progression beyond genetic mutations alone. Cancer poses a significant global health burden, with the TME and its immune components, particularly TAMs, playing crucial roles in tumor progression. Spatial transcriptomics reveals profound TME heterogeneity [30, 31]. Within the hypoxic microenvironment, stabilized HIF‐1α transcriptionally activates vascular endothelial growth factor (VEGF) to promote angiogenesis while simultaneously inducing the expression of immunosuppressive factors such as TGF‐β and adenosine [32, 33, 34]. This spatial organization creates distinct immunological zones. Spatial proteomics further demonstrated that fibrotic regions rich in α‐SMA⁺ cancer‐associated fibroblasts (CAFs) are correlated with CD163⁺ TAM accumulation and T‐cell exclusion. Conversely, perivascular zones harbor metabolically active CD8⁺ T cells [35].

Metabolic competition further shapes this landscape: tumor cells undergo metabolic reprogramming, including dysregulated lipid metabolism, to fuel their proliferation and immune evasion, depleting glucose/glutamine to induce T‐cell dysfunction. Tumor‐induced nutrient deprivation operates through active molecular mechanisms. Tumor cells export potassium ions (K⁺) via KV2.1 channels, increasing extracellular K⁺ concentrations. This directly inhibits the anti‐tumor capacity of TAMs [36, 37]. Concurrently, lactate accumulation acidifies the TME. This acidic environment activates TAMs through GPR132‐STAT3 signaling cascades [38, 39]. Notably, lipids serve not only as energy substrates but also as signaling molecules that modulate TAM polarization and function, as exemplified by oxidized LDL (oxLDL) uptake through CD36, triggering PPARγ‐dependent M2 polarization [40, 41, 42]. Molecular communication extends to ECM remodeling. CAF‐derived proteases (e.g., matrix metalloproteinases [MMPs] and LOXs) actively cleave and reorganize matrix components [43]. This enzymatic remodeling releases growth factors (e.g., TGF‐β and EGF) sequestered within the ECM, activating cognate receptor tyrosine kinases on tumor cells. These signals promote survival pathways and induce stem‐like properties. Furthermore, tumor‐derived exosomes deliver specific microRNAs (e.g., miR‐21 and miR‐155) to stromal cells, reinforcing pro‐tumorigenic signaling networks. The location of metabolites such as succinate and itaconate within different tumor areas affects macrophage function. Lipid‐laden TAMs in necrotic zones have different metabolic activities than those near blood vessels. This influences their ability to transfer lipids to metastatic cancer cells. TAM plasticity depends on metabolic flexibility. When adapting to lipid‐rich tumor environments, TAMs undergo PPARγ‐dependent gene expression changes that reinforce M2 polarization. This creates persistent metabolic memory, which remains even when cytokine signals change.

## 3 Cellular Plasticity in Cancer: Defining “Polarization”

Cellular plasticity is a defining feature of cancer progression. This refers to the reversible adaptation of cell phenotypes in response to microenvironmental cues. This adaptive reprogramming, termed “polarization,” occurs in both cancer cells and immune cells within the TME. TAMs demonstrate remarkable plasticity. They dynamically shift between pro‐inflammatory (M1‐like) and immunosuppressive, pro‐tumor (M2‐like) states influenced by tumor‐derived signals and metabolic changes. This functional spectrum extends beyond simple M1/M2 categories. Single‐cell analyses revealed that transitional TAM states expressed markers (e.g., MHC‐II⁺CD206⁺) that display both phagocytic and pro‐angiogenic functions [44, 45].

Similarly, tumor cells exhibit plasticity, notably through EMT. Driven by transcription factors, it promotes invasive properties and confers resistance to chemotherapy [46]. Individual tumor cells can also rapidly switch between glycolytic and oxidative metabolic states depending on local oxygen and nutrient levels. Epigenetic mechanisms underpin this adaptability. Hypoxia reduces TET1 enzyme levels, stabilizing repressive H3K27me3 marks at epithelial gene promoters [47]. This epigenetic “lock” maintains mesenchymal states even without sustained TGF‐β signaling [48].

**FIGURE 1:**
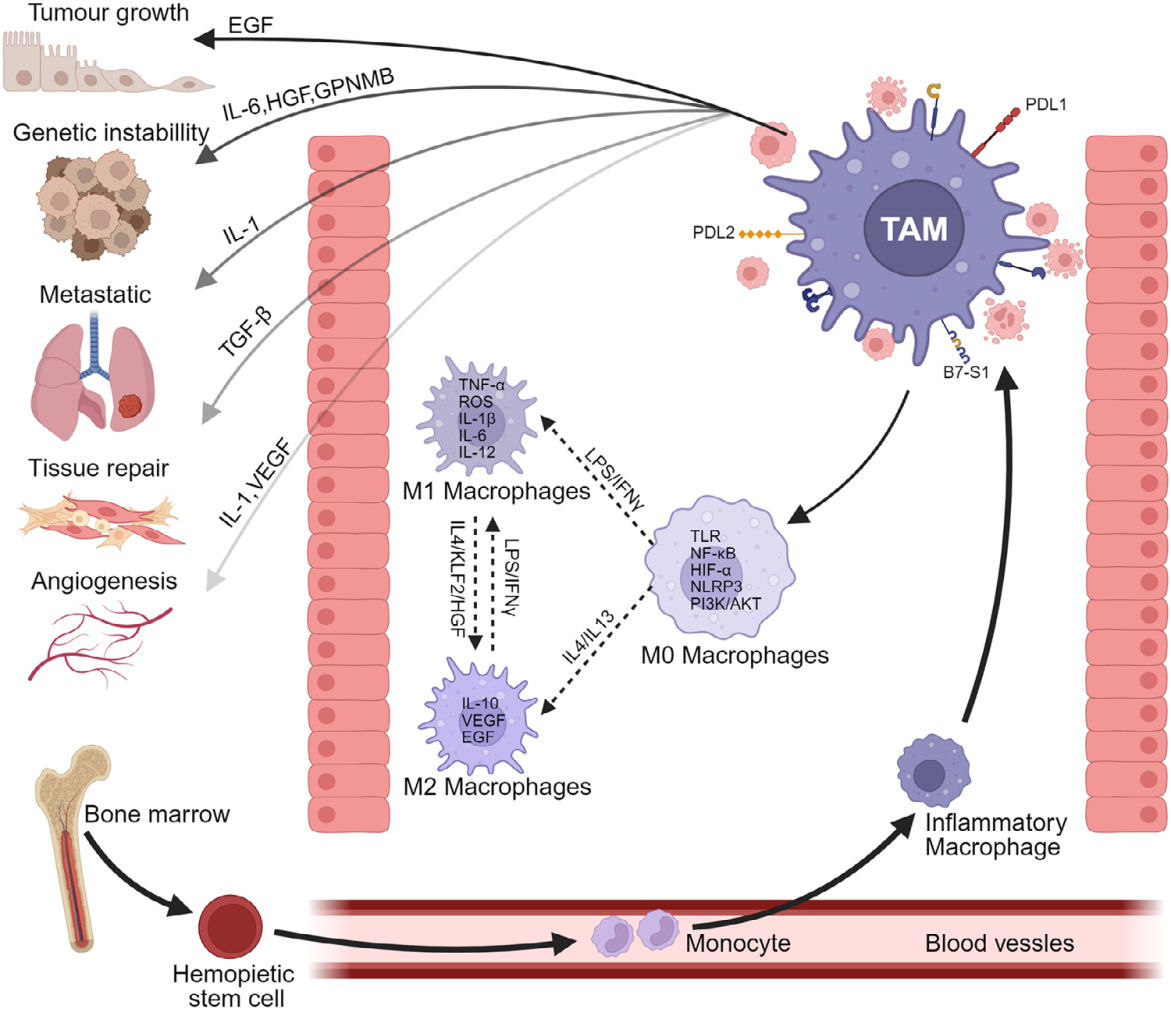
Origin and polarization of TAMs. Macrophages originate from bone marrow hematopoietic stem cells, transform into monocytes after entering the blood, and finally develop into macrophages. Under the influence of the TME, TAMs polarize and interact with tumor cells to affect tumor progression.

Metabolic reprogramming fuels cellular plasticity. Cancer cells utilize Warburg effect glycolysis and accumulate lipid droplets to support dissemination [49]. TAMs evolve from anti‐tumor M1 states in early tumors to pro‐tumor M2‐like states as the disease progresses [50, 51]. Lipid‐laden “foam” TAMs persist in necrotic areas through suppressed autophagy, acting as long‐lived sources of immunosuppressive cytokines [52]. Crucially, this plasticity is reversible. Cytokines such as interleukin‐4 (IL‐4) and IL‐13 can shift M1 TAMs toward the M2 state, whereas IFN‐γ reprograms M2 TAMs toward the M1 state [53, 54]. Figure 1 shows the development of macrophages from hematopoietic stem cells and monocytes and their polarization into different functional states within the TME. This schematic illustrates the dynamic plasticity described in the article. The functional consequences are summarized in Table 1. This adaptability exhibits hysteresis: epigenetic modifications from prior stress, such as histone lactylation, persist even after microenvironmental conditions normalize, predisposing cells to rapidly reacquire aggressive traits upon subsequent challenges [55, 56, 57].

**TABLE 1:** Phenotypic states and functional impact on TME plasticity.

Cell type	Polarization state	Key markers	Metabolic signature	Functional impact
Tumor cells	Epithelial	E‐cadherin⁺, EpCAM⁺, Keratin⁺	OXPHOS‐dominant	Proliferation, drug sensitivity, luminal confinement [46, 58]
	Mesenchymal	Vimentin⁺, N‐cadherin⁺, Fibronectin⁺	Glycolysis/lipogenesis	Invasion, metastasis, and immune escape; ECM degradation [58, 59]
	Stem‐like	CD44⁺, ALDH1⁺, OCT4⁺	FAO	Therapy resistance, dormancy, recurrence initiation [60, 61]
	Hybrid E/M	ZEB1⁺, SNAI1⁺, EpCAM^±^	Mixed metabolism	Adaptive plasticity, collective migration [46, 62]
TAMs	M1‐like (anti‐tumor)	CD80⁺, HLA‐DR⁺, iNOS⁺, TNF‐α⁺	Aerobic glycolysis	Pro‐inflammatory cytokines (TNF‐α, IL‐12); pathogen clearance [7, 12]
	M2‐like (pro‐tumor)	CD163⁺, CD206⁺, Arg‐1⁺, IL‐10⁺	OXPHOS/FAO	Tissue repair, remodeling, and tumor progression; immunosuppression [12, 52]
	Lipid‐rich TAMs	FABP5⁺, PLIN2⁺, CD36⁺	Enhanced lipid storage	Lipid droplet accumulation promoting immune tolerance; chemoresistance [63, 64]
	Immunosuppressive TAMs	VSIG4⁺, IL‐10high, TGF‐β⁺	Cholesterol efflux	T‐cell anergy via PD‐L1/CTLA‐4; regulatory T‐cell induction [65, 66]
	Pro‐angiogenic TAMs	SPP1⁺, VEGFA⁺, Tie2⁺	Glutaminyls	Neoangiogenesis; perfusion of metastatic niches [67, 68]
Other immune	Tolerogenic DCs	CD11c⁺, PD‐L1⁺, IDO⁺	Tryptophan catabolism	Regulate immune metabolic response of adipose tissue; T‐cell suppression [65, 69]
	Tumor‐associated neutrophils	CD66b⁺, ARG1⁺, MMP9⁺	Glycogenolysis	ECM remodeling; metastasis facilitation [70]
	MDSCs (PMN‐MDSC)	CD11b⁺/Ly6G⁺, ROS⁺	Fatty acid synthesis	Stimulate fat metabolism to inhibit adipocytes/angiogenesis; nitric oxide production [60, 71]

Abbreviation: FAO, fatty acid oxidation.

## 4 Tumor Cell‒TAM Crosstalk Axis: A Critical Therapeutic Nexus

Tumor cells and TAMs engage in complex reciprocal signaling within the TME. This interdependence operates through a sophisticated network involving soluble mediators, extracellular vesicles, and metabolic symbionts [72, 73, 74]. Tumor cells release signals that polarize TAMs and alter their metabolism; in turn, these adapted TAMs promote tumor cell proliferation. For example, tumor‐derived CSF‐1 recruits CCR2⁺ monocytes that differentiate into TAMs. These TAMs reciprocally secrete factors such as EGF and FGF2, which polarize TAMs back to an anti‐tumor M1‐like phenotype and inhibit monocyte migration [75]. Communication also occurs via direct physical connections. Tunneling nanotubes (TNTs) form nanometer‐scale bridges between tumor cells and TAMs, facilitating the transfer of mitochondria. This transfer rescues oxidative phosphorylation (OXPHOS) in metabolically stressed cancer cells [46, 76]. Simultaneously, TNTs shuttle miR‐21 into macrophages, suppressing PTEN expression and leading to decreased CD8^+^ T‐cell activity [77]. Furthermore, hypoxic tumor cells manipulate TAM polarization by releasing exosomal miR‐143‐3p, which suppresses RICTOR and enforces an M2 phenotype (CD206^+^/Arg‐1^+^) [78].

Metabolic interactions critically regulate this crosstalk. The accumulation of fatty acids, cholesterol efflux, and phospholipid remodeling within the TME create a feedforward loop that drives tumor progression. Under conditions of metabolic stress, TAMs upregulate arginase‐1 (ARG‐1) and differentiate into an immunosuppressive M2 phenotype. This shift, coupled with tumor cells overexpressing the anti‐phagocytic signal CD24, collectively fosters an immunosuppressive and tumor‐promoting microenvironment [79]. TAMs supply more nutrients than general nutrients. They selectively process and transfer specific lipids, such as CD36‐released free fatty acids, to fuel β‐oxidation in metastasis‐initiating tumor cells. This lipid transfer supports cancer cell survival and stem‐like properties during dissemination. Understanding these intricate metabolic and signaling pathways provides crucial insights for the development of novel immunotherapies that target the TME.

## 5 Molecular Mechanisms of Tumor Cell Polarization

The plasticity of tumor cells is dynamically orchestrated by metabolic reprogramming and signaling pathways, promoting pro‐invasive phenotypes. This section elucidates the molecular foundation of EMT activation through dysregulated lipid metabolism, such as increased β‐oxidation via lipid droplet accumulation, and key transcription factors, including Twist, Snail, and ZEB1. These findings reveal how metabolites function as signaling molecules to reshape the crosstalk between tumor cells and TAMs. These adaptive changes not only increase metastatic potential but also remodel the microenvironment through immunosuppressive factors, thereby providing a rationale for targeted intervention.

### 5.1 Phenotypic Plasticity Programs

Tumor cells exhibit remarkable plasticity, dynamically adapting to microenvironmental stressors through coordinated transcriptional and metabolic reprogramming [80]. TAMs display notable plasticity and functional heterogeneity, mirroring tumor cell evolution. While often exhibiting anti‐tumor (M1‐like) phenotypes early on, TAMs typically shift toward pro‐tumorigenic (M2‐like) states as tumors progress. This phenotypic evolution parallels the EMT that occurs in malignant cells. EMT is orchestrated by master transcriptional regulators, including Twist, Snail, and ZEB1 [58, 62, 81, 82]. These factors repress E‐cadherin expression while inducing vimentin, enhancing tumor cell motility and invasion [83].

This adaptability fundamentally stems from metabolic rewiring. The upregulation of FABP4/5 expression in tumor cells undergoing EMT leads to lipid droplet accumulation, which supplies essential membrane components for rapid proliferation [84]. Notably, migrating tumor cells exhibit increased mitochondria‒lipid droplet contacts, establishing direct channels that fuel β‐oxidation and circumvent cytosolic lipid transport [85]. Such organelle coordination demonstrates the integration of metabolic and structural changes during cellular plasticity. TAMs also display functional shifts driven by lipid metabolism. For example, FABP5‐expressing TAMs within necrotic zones accumulate triacylglycerols, which suppress MHC‐II expression and sustain immunosuppressive cytokine production [52, 63]. Spatial heterogeneity further diversifies TAM phenotypes: perivascular regions contain metabolically active immune cells, whereas fibrotic areas accumulate TAMs that promote T‐cell exclusion [86, 87]. Temporal regulation adds another layer of complexity; circadian oscillations in REV‐ERα‐driven lysosomal lipolysis fuel immunosuppressive activity during daylight hours [88].

Metabolites themselves act as signaling molecules facilitating cellular crosstalk. Succinate released by TAMs engages the SUCNR1 receptor on tumor cells, activating the PI3K/extracellular signal‐regulated kinase (ERK) pathway to drive mitogenic signaling and metastasis [89]. Conversely, tumor‐derived prostaglandin E_2_ (PGE_2_) polarizes TAMs toward M2‐like states via EP4 receptor‐dependent induction of IL‐2 [90]. This reciprocal metabolite exchange thus directly reprograms transcriptional networks, extending beyond mere nutritional support.

Therapeutic approaches are leveraging this metabolic plasticity. For example, engineered chimeric antigen receptor‐macrophages (CAR‐Ms) deficient in ACOD1 enhance tumor‐killing activity through excessive reactive oxygen species (ROS) production [91]. In contrast, neoadjuvant FAO inhibitors deplete lipid‐laden TAMs prior to checkpoint blockade, creating synergistic windows for anti‐tumor immunity [92, 93].

### 5.2 Key Signaling Pathways

Tumor cell polarization is orchestrated by interconnected signaling cascades that integrate extracellular biochemical cues with intracellular metabolic reprogramming. A key factor is dysregulated lipid metabolism driven by the PI3K/AKT/mTOR axis, which amplifies sterol regulatory element‐binding protein (SREBP) activity [94, 95]. This enhances de novo lipogenesis, supplying essential phospholipids for membrane biosynthesis during rapid proliferation [96]. Concurrently, hypoxic signaling critically influences polarization within the TME. Disrupted lipid homeostasis significantly impacts TAM function and phenotype switching. Tumor‐derived lactate activates HIF‐1α/NF‐κB signaling, inducing PD‐L1 expression on tumor cells and promoting immune evasion [97].

Lipid metabolic reprogramming directly regulates oncogenic signaling by altering metabolite flux in tumor cells. For example, cholesterol depletion mediated by ABCG1 transporter‐dependent reverse transport reduces cholesterol levels in the TME, activating the pathway to increase tumor cell lipogenesis, thereby amplifying PI3K/AKT/mTOR signaling cascades and promoting tumor proliferation [98]. Furthermore, increased FAO not only supplies energy for rapid proliferation but also stabilizes HIF‐1α via metabolites such as acetyl‐CoA, reinforcing hypoxic signaling feedback and establishing a pro‐tumor metabolic‐signaling loop [99, 100].

**FIGURE 2:**
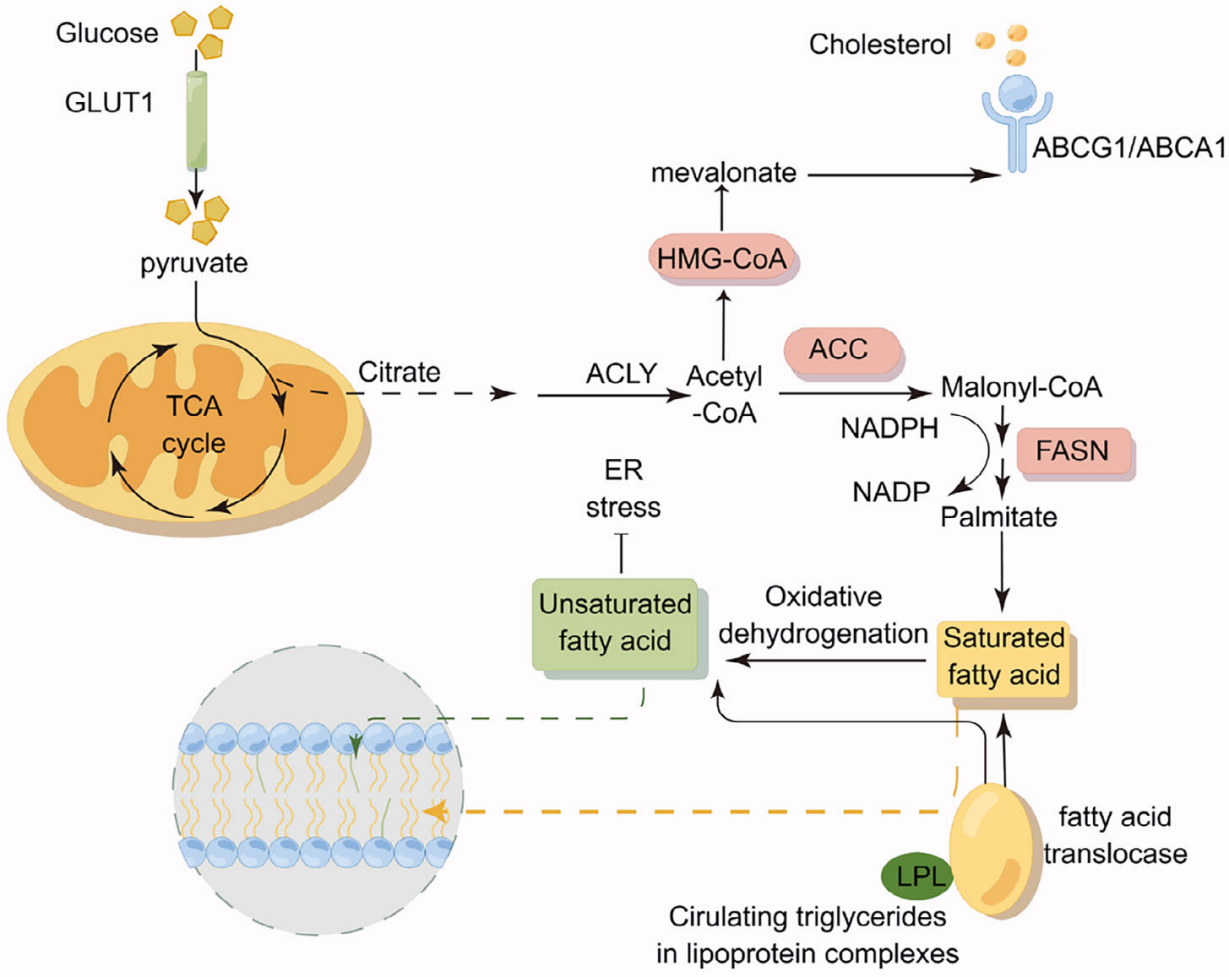
Schematic representation of intracellular lipid metabolism pathways in TAMs and tumor cells. Lipids undergo hydrolysis to yield glycerol and fatty acids, which are subsequently subjected to β‐oxidation, resulting in the production of acetyl‐CoA. This acetyl‐CoA serves as a substrate for the TCA cycle, facilitating energy production. Additionally, surplus lipids are employed in de novo fatty acid synthesis through the action of ATP‐citrate lyase (ACLY) and fatty acid synthase (FASN). Cholesterol metabolism is modulated by transporters such as ABCG1 and ABCA1, while pyruvate derived from glucose contributes to lipid biosynthesis. Lipid reprogramming enables TAMs to rapidly acquire energy, thereby supporting TAM polarization and tumor progression.

Metabolite signaling directly modulates epigenetic states. Elevated succinate levels in tumor cells are sufficient to reduce overall 5‐hydroxymethylcytosine (5hmC) accumulation and induce the transcriptional repression of EMT‐related genes, which promotes cancer progression. Figure 2 summarizes the changes in lipid metabolism that drive tumor cell polarization, revealing key processes, including FAO, de novo lipogenesis, and cholesterol efflux. Sphingolipid metabolism integrates diverse oncogenic signals. Oncogenic kinase cascades (e.g., Ras/MAPK) upregulate sphingosine kinase 1 (SPHK1) expression and activity. This generates sphingosine‐1‐phosphate (S1P), which engages cognate S1PR2 receptors in an autocrine/paracrine manner. Targeting the SphK1/S1P/PFKFB3 axis inhibits the progression of hepatocellular carcinoma [101].

### 5.3 Functional Consequences

Tumor cell polarization confers enhanced aggressiveness, therapy resistance, and immunosuppressive capabilities. Cells in a mesenchymal state exhibit significantly increased invasive potential, facilitated by increased secretion of MMP2/9 and subsequent ECM degradation [59]. Critically, stem‐like tumor cells characterized by high ALDH1 activity promote TAK1 phosphorylation and activate NF‐κB signaling, conferring resistance to chemotherapy [60]. Lipid metabolic reprogramming further endows tumor cells with immune evasion capabilities. Tumor cells secrete lipid mediators such as PGE_2_ to engage EP4 receptors on TAMs, polarizing them toward an M2‐like phenotype that suppresses T‐cell activity and promotes immune tolerance [102]. Concurrently, disrupted cholesterol metabolism depletes lipid rafts in TAM membranes, impairing antigen presentation and accelerating tumor immune escape. With respect to therapy resistance, upregulation of the fatty acid‐binding protein FABP5 reduces β‐oxidation rates, leading to lipid droplet accumulation in TAMs and fostering an immunosuppressive microenvironment; inhibiting FABP5 reverses lipid accumulation and enhances chemosensitivity [64]. Elevated expression of CD36—a key fatty acid transporter—intensifies tumor cell reliance on the lipid supply from TAMs, and targeting CD36 disrupts fatty acid transfer while inhibiting metabolic adaptation in metastatic tumor cells [61]. This resistance is further propagated through extracellular vesicle exchange: cisplatin‐treated stem cells release Annexin A6⁺ exosomes that transfer multidrug resistance protein 1 (MRP1) to neighboring tumor cells, markedly reducing intracellular drug accumulation [103].

Tumor‐derived metabolites actively drive progression and immune evasion. PGE_2_, which is synthesized from arachidonic acid (AA), plays a dual role: it stimulates tumor cell migration while simultaneously polarizing TAMs toward immunosuppressive M2‐like phenotypes via EP4 receptor signaling [90, 104]. When tumor cells develop a mesenchymal phenotype, they become more dependent on external lipids. This makes them sensitive to blocking TAM‐mediated fatty acid transfer. CD36 inhibition can exploit this vulnerability.

## 6 Molecular Mechanisms of TAM Polarization

The polarization of TAMs presents a complexity that surpasses the conventional M1/M2 classification. This heterogeneity manifests both spatially and temporally within the TME. Comprehensive single‐cell profiling of TAM subsets elucidates the role of hypoxia, exosomal miRNAs, and lncRNA signaling pathways in epigenetically driving immunosuppressive phenotypes. Moreover, the polarization states of TAMs exhibit a degree of persistence and plasticity, referred to as “metabolic memory,” which affects their potential for reversal. This intrinsic plasticity positions TAM polarization as a pivotal target for therapeutic intervention.

### 6.1 TAM Heterogeneity: Moving Beyond M1/M2

The classical dichotomy of M1/M2 macrophages fails to capture the functional spectrum of TAMs, with recent single‐cell technologies revealing unprecedented heterogeneity. The classical classification of TAMs can be divided into two categories: classically activated macrophages (M1) and alternatively activated macrophages (M2). However, spatial transcriptomics identifies at least seven subsets, including SPP1⁺ pro‐angiogenic TAMs in hypoxic regions, VSIG4⁺ immunosuppressive TAMs in invasive fronts, and lipid‐laden FABP5⁺ TAMs within necrotic zones [105]. These functionally distinct subsets, characterized by unique metabolic profiles and pro‐tumorigenic functions, are systematically summarized in Table 2. Importantly, macrophage polarization markers are usually expressed at different levels in different types of macrophages, and other kinds of macrophages can undergo mutual transformation under specific conditions. For example, IL‐10 induces FABP5⁺ lipid‐rich TAMs that store triacylglycerols while suppressing MHC‐II expression [52]. Transcriptional profiling reveals distinct enhancer landscapes associated with these spatially defined subsets, indicating that epigenetic imprinting by the local microenvironment stabilizes their functional states. Temporal heterogeneity adds complexity: intravital imaging reveals circadian oscillations in TAM lipid metabolism, with REV‐ERα‐driven lysosomal lipolysis peaking at night to fuel daytime immunosuppressive functions [88]. The metabolic specialization of macrophages across tumor locations and stages shapes their function. Macrophages in dead tissue areas, which are rich in lipids and highly express CD36 and FABP5, transfer fatty acids to nearby tumor cells. This finding directly supports metastasis.

**TABLE 2:** Functionally defined TAM subsets in human carcinomas.

Subset	Key markers	Metabolic profile	Pro‐tumor function
Immunosuppressive	VSIG4⁺, IL‐10high, TGF‐β⁺	Cholesterol efflux	Induces T‐cell exhaustion via PD‐L1/IDO; recruits Tregs [65, 66]
Pro‐angiogenic	SPP1⁺, VEGFA⁺, Tie2⁺	Glutaminyls	Forms abnormal vasculature (leakage ≥40%); mediates anti‐VEGF resistance [67, 68]
Lipid laden	FABP5⁺, CD36⁺, PLIN2⁺	Enhanced lipid storage	Promotes chemoresistance and immune tolerance via lipid droplet accumulation [52, 63]
ECM remodeling	MMP9⁺, LOXL2⁺, FN1⁺	Glycolysis	Degrades basement membrane; activates EMT through TGF‐β release [43, 70]
Iron recycling	FTL⁺, CD71⁺, HMOX1⁺	Ferritin synthesis	Protects tumor cells from ferroptosis; scavenges ROS [63, 106]
Antigen presenting	HLA‐DR⁺, CD74⁺, LAMP3⁺	Amino acid catabolism	Fulfills adaptive immunity as antigen presenting cells; induces T‐cell anergy [65, 107]
Hypoxia adapted	CA9⁺, GLUT1⁺, BNIP3⁺	Aerobic glycolysis	Produces pro‐inflammatory cytokines; secretes ADM to enhance metastasis [33]
Phagocytic deficient	SIRPα⁺, CD47⁺, MERTK⁺	β‐Oxidation process	Engulfing pathogens impairment; promotes “don't‐eat‐me” signaling [79]
Neurotrophic	NGF⁺, BDNF⁺, TrkB⁺	Ketone body utilization	Repairing and renewing tissues; induces perineural invasion [108]
Senescence associated	p16⁺, CCL2⁺, CXCL8⁺	SASP secretion	Creates pro‐metastatic chronic inflammation [102]
Interferon responsive	STAT1⁺, IRF7⁺, ISG15⁺	Tryptophan depletion	Correlates with anti‐PD‐1 resistance [69]
Lipid synthesizing	FASN⁺, SCD1⁺, ACLY⁺	De novo lipogenesis	Provides phospholipids for tumor membrane biosynthesis; secretes PGE_2_ [90, 109]

### 6.2 Recruitment and Polarization Cues

TAM education begins when tumor‐derived signals recruit and polarize monocytes toward immunosuppressive states. In early tumor stages, sufficient oxygen predominantly maintains M1‐like TAMs. During this phase, the CCL2/CCL5 chemokines attract CCR2⁺ monocytes, which differentiate into inflammatory macrophages under IFN‐γ and lipopolysaccharide (LPS) stimulation. As tumors progress, however, chronic oxygen and nutrient deprivation drives metabolic reprogramming, which shifts most TAMs toward M2‐like phenotypes [110].

Hypoxia stabilizes HIF‐1α, triggering adenosine and TGF‐β secretion [111]. This suppresses IRF5 while activating PPARγ and redirecting glucose through the pentose phosphate pathway to generate NADPH for lipid biosynthesis [112]. Concurrently, ABCG1‐mediated cholesterol efflux promotes M2 polarization: tumor cells export cholesterol via ABCA1/ABCG1 transporters. This depletion activates AMPKα and metabolic reprogramming [112]. By delivering lipids and miRNAs, tumor‐derived exosomes further reinforce polarization. Notably, miR‐21‐5p silences PTEN in TAMs, enhancing PI3K/AKT signaling. It is a critical metabolic adaptation that stabilizes the M2 state [113]. Lactic acid provides another layer of regulation; as lactate activates GPR132 receptors on TAMs, it promotes the polarization of TAMs toward the M2 phenotype, thereby inhibiting tumor growth and angiogenesis [114]. Prolonged exposure to tumor‐derived lipids, particularly through the CD36‐mediated uptake of oxidized LDL, sustains TAMs in an immunosuppressive state. This is facilitated by PPARγ‐driven epigenetic modifications that enhance the expression of immunosuppressive genes. These modifications result in enduring metabolic adaptations that are resistant to reprogramming efforts.

### 6.3 Intracellular Signaling Drivers

Conserved molecular circuits within TAMs translate microenvironmental signals into polarized phenotypes. Macrophage metabolism is dynamically regulated, particularly through enhancing the interaction between hexokinase 2 (HK2) and ITPR3 (the main Ca^2+^ channel on the endoplasmic reticulum). This interaction reduces Ca^2+^ levels in the cytoplasm and mitochondria, thereby inhibiting the activation of the MAPK, STAT1, and the NLRP3 inflammasome [115]. Ultimately, it hinders the polarization of M1‐type macrophages, promoting tumor growth.

The current consensus suggests that IL‐4/IL‐13 signaling activates STAT6, shifting TAMs toward the M2 phenotype. The resulting epigenetically lock TAMs into M2‐like states. Mitochondrial stress responses integrate these signals: lipid overload triggers the mitochondrial unfolded protein response (mtUPR), which activates ATF5 to increase mitophagy [116]. Cholesterol metabolism directly shapes TAM function. When ABCG1 exports cholesterol, it disrupts membrane “lipid rafts.” This weakens pro‐inflammatory IFN‐γ signaling while increasing pro‐tumor IL‐4 signaling, locking TAMs in the M2 state. Animal studies have shown that blocking ABCG1 restores anti‐tumor TAM function [71]. Post‐translational modification mechanisms further fine‐tune these responses. Lipid droplet buildup drives TAM immunosuppression. In liver cancer, elevated FABP5 protein slows fatty acid breakdown, causing lipid droplets to accumulate. These droplets act as “command centers,” gathering immunosuppressive molecules that help tumors evade immunity by increasing PD‐L1 expression on Treg cells [117]. For example, macrophage responses to repair signals involve reduced phosphorylation of PPARγ at threonine 166 (T166). This post‐translational modification enables PPARγ to bind to regulatory regions of fatty acid synthase (FAS) genes, driving intracellular lipid synthesis and accumulation. This phosphorylation‐dependent mechanism has emerged as a critical regulator of M2 macrophage function through lipid metabolic reprogramming [118]. Metabolic intermediates directly enable macromolecule functions. In pro‐inflammatory TAMs, ACD40 activation activates FAO and glutamine metabolism, which promotes pro‐inflammatory gene expression and anti‐tumor phenotypes in macrophages through ATP citrate lyase‐dependent epigenetic reprogramming [119]. Conversely, ABCA1‐driven cholesterol depletion in the cell membrane and disruption of lipid rafts enhance IL‐4 signaling and impair IFN‐γ signaling, creating an M2‐polarizing feedforward loop [120]. The metabolic status of TAMs determines their signaling activity, particularly through lipid droplet accumulation and FAO capacity. In lipid‐loaded TAMs, PPARγ signaling intensifies because transcription factors and metabolites accumulate at lipid droplets. This process strengthens the immunosuppressive function.

### 6.4 Pro‐Tumorigenic Functions

Polarized TAMs drive tumor progression through multiple mechanisms involving metabolic crosstalk and secretory programs. M2‐like TAMs secrete anti‐inflammatory cytokines, including IL‐10, TGF‐β, VEGF, and EGF, establishing an immunosuppressive TME. Within this niche, TGF‐β suppresses CD8⁺ T‐cell function by inhibiting granzyme B production, whereas VEGF‐A promotes abnormal angiogenesis and vessel permeability [67, 121]. Beyond cytokine secretion, TAMs create localized nutrient sinks. TAM‐produced PGE_2_ fuels immune escape. This lipid molecule enhances Treg suppression while “brainwashing” nearby TAMs into the M2 type. Blocking PGE_2_ in thyroid cancer disrupts this vicious cycle [102]. IDO1‐expressing TAMs deplete tryptophan, increasing kynurenine levels. This metabolite acts as an endogenous ligand for the aryl hydrocarbon receptor (AhR) on T cells, driving PD‐1 upregulation and functional exhaustion independently of checkpoint ligands. Concurrently, kynurenine‐mediated AhR activation expands immunosuppressive regulatory T‐cell networks [65].

Polarized TAMs also critically contribute to tumor immunosuppression, angiogenesis, and metabolic reprogramming. Lipid‐laden TAMs transfer fatty acids via CD36 to adjacent tumor cells, fueling β‐oxidation and supporting the survival of metastasis‐initiating cells [89]. Drug‐resistant tumors “tame” TAMs. Cisplatin‐resistant lung cancer cells signal TAMs to increase FABP5 protein levels. This allows TAMs to stockpile lipid droplets as “shields” for tumor cells. Inhibiting FABP5 removes this protection [64]. TAMs provide active metabolic support rather than passive assistance. They selectively sort and distribute specific lipids to create a specialized environment. This environment favors more aggressive cancer cells with an increased ability to spread. Direct organelle transfer further amplifies TAM support: TNT‐induced TAMs exhibit enhanced pro‐tumorigenic properties. TNT‐mediated transfer of tumor‐derived AA reprograms TAMs through PI3K‒AKT activation, promoting immune suppression and tumor progression [122].

ECM remodeling is another key TAM function. TAMs release sequestered TGF‐β and EGF‐like growth factors. TAMs increase the MMP, and the MMP degrades interstitial collagen, increases collagen synthesis and organization, and remodels the TME, which is conducive to tumor cell invasion [70]. Ultimately, TAM‐derived mediators such as PGE_2_ and adenosine engage EP2/A2A receptors on Tregs, expanding immunosuppressive networks that confer resistance to anti‐PD‐1 therapy [66].

## 7 Bidirectional Crosstalk During Polarization

The interaction between tumor cells and TAMs constitutes a self‐perpetuating, deleterious cycle facilitated by metabolic symbiosis. This section explores the feedforward signaling networks that underpin this interaction. Central mechanisms include the exchange of metabolites such as lactate, lipids, and succinate; the transfer of organelles by TNTs; and the secretion of immunosuppressive factors such as IDO1 and PGE_2_. Additionally, the acidic TME promotes immune evasion by inducing specific histone modifications, such as lactylation‐mediated suppression of retinoic acid receptor‐γ (RARγ), and instigating mitochondrial stress, particularly through the ACOD1‒itaconate axis. The spatial organization of metabolism, which varies between necrotic core regions and perivascular zones, further strengthens this pathological collaboration.

### 7.1 Tumor Cell → TAM Polarization

Tumor cells reprogram the metabolism of neighboring macrophages through diverse signaling mechanisms, establishing pro‐tumor phenotypes. Soluble mediators in the TME, including cancer cell‐derived exosomes, activate TLR2 on macrophages. This engagement triggers the NF‐κB and HIF‐1α signaling pathways, leading to increased transcription of glycolytic enzymes and increased lactate export [123]. Hypoxic gradients further stabilize HIF‐1α, promoting macrophage expression of VEGF and ARG‐1, which enhances immunosuppression and angiogenesis [68].

Tumor‐derived signals systematically shift macrophage metabolism from OXPHOS toward glycolysis [124]. This shift leads to the upregulation of enzymes such as pyruvate kinase M2 (PKM2) and lactate dehydrogenase A (LDHA) [125]. In addition to their metabolic roles, these enzymes sustain pro‐tumor functions through secondary “moonlighting” activities. Critically, metabolic byproducts such as lactate also act as signaling molecules. Lactate induces histone lactylation in macrophages, repressing anti‐tumor genes (e.g., RARγ) while activating the transcription of immunosuppressive IL‐6 [126]. Furthermore, cancer‐derived succinate activates SUCNR1 receptors on macrophages, polarizing them toward metastasis‐promoting states via the HIF‐1α and PI3K signaling cascades [127]. This metabolic reprogramming extends to lipid metabolism. Tumor‐derived extracellular vesicles deliver fatty acids to macrophages via CD36 receptors, driving lipid droplet accumulation and fueling FAO [128]. This FAO is essential for TAM survival and function within the TME. Tumor cells further manipulate TAMs by forcing them to accumulate excessive lipids. This lipid overload drives TAMs into an immunosuppressive state characterized by abundant lipid droplets. In this altered state, TAMs act as metabolic reservoirs, storing fatty acids that are later transferred back to tumor cells to fuel their dissemination.

### 7.2 TAM → Tumor Cell Polarization

TAMs utilize metabolic mechanisms to directly influence tumor cell behavior and promote malignancy. In addition to supplying energy substrates, metabolites derived from TAMs generate mitochondrial reactive oxygen species (mtROS). This mtROS inflicts DNA damage on adjacent cancer cells. Such damage accelerates mutagenesis and promotes genomic instability, ultimately driving tumor evolution and enhancing heterogeneity. mtROS are generated within TAMs due to disruptions in the tricarboxylic acid (TCA) cycle. This occurs particularly through aconitate decarboxylase 1 (ACOD1)‐driven itaconate production, which inflicts DNA damage on neighboring cancer cells [129]. This accelerates mutagenesis and contributes to genomic instability. Furthermore, lipid‐laden TAMs export cholesterol and free fatty acids. Tumor cells scavenge these molecules, activating proliferative signaling pathways; for example, cholesterol uptake fuels androgen receptor signaling in prostate cancer [130]. In pancreatic malignancies, TAM‐derived IL‐1β activates NF‐κB signaling in tumor cells, increasing their invasive potential and metastatic dissemination [108].

Metabolite exchange is central to this symbiotic relationship [131, 132]. Succinate expelled by TAMs binds to succinate receptor 1 (SUCNR1) on cancer cells, triggering mitogenic signaling via the PI3K and ERK pathways [133]. Additionally, PGE_2_ synthesized by TAMs induces epigenetic reprogramming in cancer cells. This occurs through PGE_2_‐mediated upregulation of ubiquitin‐like with PHD and RING finger domains 1 (UHRF1), which promotes hypomethylation of oncogenic promoters, such as the colony‐stimulating factor 1 (CSF1) gene [134]. Through this bidirectional metabolic crosstalk, TAMs critically support tumor growth, survival, and the development of heterogeneity. In addition to fueling tumor cells, the selective transfer of specific fatty acids from TAM lipid droplets also activates oncogenic signaling pathways, such as the PI3K/AKT pathway. This direct link between TAM metabolism and oncogenic signaling enhances tumor cell aggressiveness.

### 7.3 Amplification Circuits

Reciprocal interactions between tumor cells and TAMs establish self‐reinforcing metabolic loops that consolidate immunosuppression and drive disease progression. TAMs stimulate cancer cells to transcribe CSF1. CSF1 then binds to its receptor (CSF1R) on macrophages, amplifying PPARγ‐driven lipid metabolism.

Lactate acts as another key mediator in this crosstalk. Tumor cells export lactate via monocarboxylate transporter 4 (MCT4), which is subsequently imported by TAMs through monocarboxylate transporter 1 (MCT1) [135]. This lactate fuels histone lactylation within TAMs [136]. Epigenetic modifications suppress anti‐tumor genes such as RARγ while activating immunosuppressive IL‐6/STAT3 signaling.

Microenvironmental acidification further stabilizes this symbiotic relationship. TAM‐synthesized polyamines buffer intracellular pH, enabling sustained macrophage function under acidic conditions while simultaneously inhibiting cytotoxic T‐cell activity. Furthermore, TAMs secrete MMPs, often induced by CD40 agonism, which degrades collagen [137]. This degradation releases arginine‐rich fragments that, in turn, feedback to increase ARG‐1 activity within macrophages. Consequently, local arginine pools essential for T‐cell receptor signaling are depleted. These spatially organized positive feedback loops progressively reinforce metabolic cooperation between tumor cells and TAMs, rendering it increasingly self‐sustaining.

### 7.4 Metabolic Crosstalk as the Molecular Engine

In addition to independent metabolic pathways, the interplay between lipid, amino acid, and glucose metabolism forms a molecular engine that fuels tumor progression. Lipid metabolism directly regulates glucose utilization: CD36‐mediated fatty acid uptake in TAMs suppresses glycolysis while enhancing OXPHOS, creating an M2‐favoring metabolic state that provides lactate and ketone bodies to fuel neighboring tumor cells [138]. Conversely, tumor‐derived glutamine depletion forces TAMs to break down lipids through β‐oxidation, releasing acetyl‐CoA, which stabilizes HIF‐1α in cancer cells and promotes their invasive potential [139]. Cholesterol metabolism further bridges amino acid signaling: ABCG1‐driven cholesterol efflux from TAMs disrupts lipid rafts on tumor cells, amplifying mTORC1 sensitivity to extracellular leucine and activating pro‐growth ribosomal biogenesis [140]. This crosstalk establishes a vicious cycle in which phospholipid‐derived mediators such as PGE_2_ (synthesized from AA in TAMs) not only polarize additional macrophages toward the M2 phenotype but also inhibit glucose uptake in cytotoxic T cells, simultaneously promoting immunosuppression and nutrient monopolization [141]. Critically, these metabolic interactions involve spatially organized lipid‐loaded TAMs in necrotic zones that export FABP5‐bound fatty acids to hypoxic tumor regions, where they are oxidized to support ATP production for PD‐L1 glycosylation, linking lipid reprogramming directly to immune checkpoint activation.

### 7.5 Pathological Outcomes

Metabolic cooperation between tumor cells and TAMs contributes significantly to adverse clinical outcomes, including metastasis, therapy resistance, and immune exhaustion. Lactate‐driven histone lactylation in TAMs promotes the formation of a pre‐metastatic microenvironment [142, 143]. This epigenetic modification suppresses chemokines, impairing dendritic cell recruitment and cytotoxic T‐cell infiltration into distant organs [144, 145].

Furthermore, lipid‐loaded TAMs facilitate metastatic spread by secreting cathepsin B, which remodels the extracellular matrix to enable cancer cell invasion [146, 147]. Importantly, TAMs residing in the pre‐metastatic microenvironment undergo metabolic reprogramming that enhances their lipid storage capacity. This creates a lipid‐rich reservoir crucial for fueling the initial colonization and subsequent outgrowth of disseminated tumor cells. Therapy resistance develops through complementary mechanisms. Competition for glutamine depletes this essential amino acid in TAMs, reducing their phagocytic capacity and allowing cancer cells to evade elimination. Additionally, glycolytic enzymes in TAMs, such as PKM2, upregulate immune checkpoint molecules such as PD‐L1 on tumor cells, shielding them from T‐cell attack [148, 149]. These pathophysiological consequences highlight why disrupting the metabolic crosstalk between tumors and TAMs represents a promising therapeutic strategy for improving cancer outcomes.

## 8 Therapeutic Target Strategies

Disrupting the metabolic symbiosis between tumors and TAMs necessitates comprehensive strategies that target this critical crosstalk. Current research focuses on three key approaches: the use of small‐molecule inhibitors to block lipid transfer and disrupt the energy supply, the use of nanocarriers designed for specific delivery to reprogram TAM metabolism, and the development of engineered CAR‐M‐cell therapies to increase anti‐tumor activity. Importantly, sequential treatment regimens, such as the administration of FAO inhibitors before PD‐1 blockade, can effectively counter microenvironmental adaptation. Furthermore, simultaneously targeting both lipid acquisition and utilization pathways induces synthetic lethality within this symbiotic relationship.

### 8.1 Targeting Tumor Cell Plasticity

Targeting the metabolic plasticity of tumor cells represents a fundamental strategy in modern cancer therapy, aiming to disrupt the adaptive mechanisms of malignancies and render them vulnerable to destruction. Small‐molecule inhibitors, such as glutamine antagonists (e.g., JHU083), exploit the dependence of tumor cells on specific nutrient utilization pathways. These agents impair nucleotide synthesis and redox balance while simultaneously enhancing immune recognition by exposing previously hidden antigenic epitopes [150]. These inhibitors capitalize on synthetic vulnerabilities inherent to cancer metabolism. For example, tumors adapted to hypoxia exhibit heightened reliance on serine biosynthesis enzymes such as phosphoglycerate dehydrogenase (PHGDH) [151]. Pharmacological inhibition of PHGDH can induce lethal metabolic stress in these malignancies. Crucially, metabolic interventions also remodel the TME by altering metabolite secretion. Glutamine restriction reduces 2‐hydroxyglutarate secretion in isocitrate dehydrogenase (IDH)‐mutant gliomas, thereby diminishing immunosuppression in TAMs [152].

Emerging therapeutic strategies further target epigenetic reprogramming enzymes activated by oncometabolites. These approaches reverse DNA hypermethylation events that silence tumor suppressor genes. By systematically dismantling the metabolic foundations that sustain tumor heterogeneity, such therapies transform the TME from a protective niche into a hostile environment for malignant cells. Disrupting the dependence of tumor cells on TAM‐derived lipids, particularly by blocking fatty acid uptake, impairs the survival of metastasis‐initiating cells. This approach offers a promising strategy to target tumor cell plasticity.

### 8.2 Reprogramming TAMs

Reprogramming TAMs from immunosuppressive to immunostimulatory states targets key metabolic regulators. Pattern recognition receptor agonists, especially TLR9 ligands such as CpG oligonucleotides, restructure macrophage mitochondrial metabolism [153]. This shift toward oxidative phosphorylation enhances their ability to phagocytose antibody‐opsonized tumor cells, even in the presence of “don't‐eat‐me” signals. Similarly, TREM2 blockade disrupts lipid sensing in lipid‐laden TAM subsets, inhibiting PPARγ‐driven FAO [154]. Targeted inhibition of enzymatic pathways also shows promise: blocking ACOD1 reduces itaconate accumulation, reactivating inflammatory responses suppressed by nuclear factor erythroid 2‐related factor 2 and restoring phagocytic function [91]. Clinical‐stage compounds such as the glutamine antagonist prodrug DRP‐104 demonstrate dual efficacy: they starve tumor cells while triggering glycolytic bursts in TAMs that enhance antigen presentation [155, 156, 157]. This metabolic reprogramming transforms TAMs from tumor supporters into effectors of anti‐tumor immunity. Engineered macrophage adoptive therapies, such as CAR‐M cells with ACOD1 deletion, exploit metabolic rewiring to increase ROS production and enhance tumor‐killing activity. This approach represents a potential strategy to disrupt the metabolic interplay between TAMs and tumor cells.

### 8.3 Combination Therapies

Synergistic therapeutic strategies combining metabolic modulators with immunotherapies can overcome compensatory resistance mechanisms within the TME. For example, COX‐2 inhibitors such as celecoxib reduce PGE_2_‐mediated immunosuppression by downregulating PD‐L1 expression on TAMs and suppressing itaconate production [158]. This reversal of CD8⁺ T‐cell exhaustion enhances the efficacy of PD‐1/PD‐L1 checkpoint blockade, particularly in immunologically cold tumors. Simvastatin (HMG‐CoA inhibitor) plus paclitaxel increases overall survival in non‐small cell lung cancer through LXR/ABCA1‐dependent TAM repolarization and EMT suppression [159]. Sequencing is critical for metabolic interventions: a neoadjuvant FAO inhibitor (perhexiline) is administered 72 h before anti‐PD‐1‐depleted lipid‐laden TAMs, creating an “immune‐permissive window” that enhances T‐cell infiltration more effectively than concurrent dosing in melanoma [160]. This temporal sequence allows the TME to reset from a lipid‐rich, immunosuppressive state to one more receptive to T‐cell infiltration and activation before checkpoint blockade unleashes the immune response. Immune checkpoint inhibitors synergize with metabolic modulators: CD36 blockade combined with anti‐PD‐1 therapy reduces lipid‐laden TAMs and increases the CD8⁺/Treg ratio in melanoma, overcoming anti‐PD‐1 resistance [161]. Subsequent targeting has proven critical; a neoadjuvant CCR2 inhibitor (PF‐04136309) depletes TAMs, inhibiting GOLM1‐mediated colorectal cancer (CRC) metastasis in residual tumor cells [162, 163]. Cancer cell plasticity, stromal metabolite support, and T‐cell dysfunction convert transient responses into durable remissions. Clinical validation is emerging, with cancer trials showing doubled overall survival for COX‐2/anti‐PD‐L1 combinations versus monotherapy [164, 165]. Targeting TAM lipid metabolism through CD36 inhibition combined with agents that impair tumor cell lipid utilization (e.g., FAO inhibitors) generates synthetic lethality. This dual approach simultaneously starves lipid‐producing tumor cells and reprograms TAMs, significantly improving anti‐tumor efficacy in preclinical models.

### 8.4 Nanotechnology Solutions

Nanoscale delivery platforms provide precise tools to manipulate TAM metabolism while minimizing off‐target effects [166, 167]. These systems exploit the unique pathophysiology of the TME to increase specificity. Microparticles and nanoparticles passively accumulate in tumors through the enhanced permeability and retention effect, where phagocytosis by TAMs facilitates localized metabolic reprogramming [168, 169].

For example, liposomal clodronate depletes immunosuppressive TAMs, whereas metformin‐loaded macrophage‐derived microparticles overcome PD‐1 resistance and increase the accumulation of CD8^+^ T cells in tumor tissues [170]. pH‐responsive nanoparticles further leverage the acidic TME, releasing IL‐12 payloads at tumor sites to reprogram TAMs while avoiding systemic inflammation [170]. In addition to passive targeting, surface engineering with scavenger receptor (SR) ligands—including anti‐CD206 peptides or MARCO‐binding motifs—actively directs nanocarriers to TAMs [171]. These constructs deliver metabolic inhibitors such as diacylglycerol O‐acyltransferase (DGAT) blockers to disrupt lipid droplet formation, reducing the frequency of pro‐tumor TAMs in cancer.

DNA‐based nanodevices represent an emerging approach: cysteine protease inhibitors delivered via oligonucleotides target TAM lysosomes through SR‐mediated endocytosis, inhibiting metastasis driven by cathepsins. In adoptive cell therapy, nanotechnology integrates genetic engineering to metabolically augment CAR‐Ms. CRISPR‐Cas9 nanoparticle delivery enables Acod1 deletion in CAR‐T cells. The anti‐tumor effect of ACOD1^−^/^−^ MSLN‐CAR‐iMAC can be further enhanced by combination with immune checkpoint inhibitors. Similarly, lipid nanoparticles encapsulating siRNAs silence key metabolic regulators in TAMs, synergizing with chemotherapy [79]. Nanoparticles functionalized with CD206‐targeting ligands can selectively deliver siRNA to TAMs. This approach effectively silences key components of the lipid shuttle system, avoiding systemic toxicity while reprogramming TAMs toward a pro‐inflammatory, anti‐tumor phenotype.

Despite promising preclinical results, clinical translation requires careful optimization of biodistribution and payload kinetics. Ongoing trials (e.g., NCT04660929 evaluating CAR‐Ms) will determine whether nanoscale precision can overcome TAM metabolic heterogeneity, potentially advancing a new era of localized immunometabolism treatments [172, 173].

### 8.5 Targeting the Metabolic Crosstalk Nexus

Targeting the metabolic interplay between tumors and immune cells represents a key strategy for disrupting the reciprocal metabolic reprogramming of malignant cells and TAMs. Lipid metabolism drives TAMs toward a pro‐tumor M2 state through three linked pathways: fatty acid accumulation, cholesterol efflux, and phospholipid signaling. Studies in hepatocellular carcinoma models have demonstrated that CD36‐mediated fatty acid uptake by TAMs promotes IL‐1β secretion via FAO. This process directly enhances tumor cell migration and immune evasion [93]. Specifically, cancer cells export cholesterol via transporters such as ABCG1, depleting cholesterol in the TME. This depletion reprograms TAMs toward M2 polarization and accelerates tumor progression [174]. Phospholipid metabolites further support this symbiotic relationship. For example, AA and its derivative PGE_2_ suppress pro‐inflammatory M1 markers in TAMs, such as TNF‐α and IL‐6, while increasing the levels of immunosuppressive M2 markers, such as IL‐10 and ARG‐1 [109].

Therapeutically, strategies inhibiting multiple pathways show promise in breaking this metabolic alliance. CD36 blockade reduces lipid droplet accumulation in TAMs and reverses their M2 polarization. Combining this combination with FAO inhibitors impairs metastatic adaptation in lymph nodes [175]. Similarly, targeting FABP5, a lipid chaperone upregulated by tumor‐derived IL‐10, decreases lipid storage in TAMs and restores β‐oxidation rates, countering chemotherapy resistance in hepatocellular carcinoma [64]. Simvastatin offers another approach by activating the LXR/ABCA1 axis. This remodels the TME, disrupts cholesterol‐rich lipid rafts in TAMs, forces their repolarization toward the M1 phenotype, and suppresses EMT [159].

These findings illustrate how intercepting critical metabolic communication points can transform TAMs from tumor supporters into immunological allies. These findings provide a rationale for combining lipid metabolism modulators with immunotherapies.

### 8.6 Clinical Challenges

The clinical translation of therapies targeting TAM metabolism faces significant hurdles. TAM heterogeneity, arising from diverse origins, tissue‐specific signals, and dynamic interactions within the TME, complicates intervention strategies [176, 177, 178]. Single‐cell analyses revealed spatially segregated TAM subpopulations with distinct metabolic profiles. This spatial and temporal metabolic plasticity can undermine treatment efficacy, as inhibitors targeting one pathway, such as glutamine antagonism, may inadvertently activate compensatory mechanisms in resistant subpopulations.

Furthermore, achieving localized TAM modulation without systemic side effects remains a challenge for conventional drug delivery. Small‐molecule inhibitors such as COX‐2 blockers or glutamine antagonist prodrugs disrupt cancer cell metabolism but simultaneously alter systemic immune function, potentially triggering autoimmune complications or opportunistic infections. Nanodelivery systems targeting macrophage SRs, such as CD206 or MARCO, offer promising alternatives. For example, pH‐sensitive nanoparticles loaded with DGAT inhibitors or metformin can release their payload selectively within acidic tumor regions. This approach aims to reprogram TAM lipid metabolism specifically within the TME while sparing macrophages in healthy tissues [179].

Despite these advances, pharmacodynamic challenges persist, particularly in monitoring real‐time metabolic responses within TAMs. Non‐invasive imaging techniques such as PET‒MRI, which target TAM‐specific metabolites such as itaconate or succinate, could help guide dose optimization. In the clinic, designing effective combination regimens requires careful sequencing. While CSF1R inhibitors can deplete immunosuppressive TAMs, they may paradoxically upregulate PD‐L1 expression in the remaining population. Therefore, rational clinical trial design must integrate spatial metabolomics data and immune monitoring to navigate the complex balance between reprogramming beneficial TAMs and eliminating tumor‐promoting subsets. The metabolic heterogeneity of TAMs—particularly variations in lipid droplet composition between distinct tumor regions, such as the necrotic core and invasive front—requires biomarker‐guided patient stratification. This spatial diversity further demands therapies with precisely targeted delivery mechanisms.

## 9 Clinical Translation and Future Perspectives

Clinical translation strategies targeting TAMs face challenges because of their metabolic heterogeneity and delivery barriers. To address these issues, spatial metabolomics techniques offer the potential for improved patient stratification. Promising strategies to overcome fibrotic resistance include engineered bacteria designed for targeted delivery of agents such as histone deacetylase (HDAC) inhibitors and artificial intelligence (AI)‐designed nanocarriers. Future progress will need to integrate single‐cell metabolomics with organoid models to develop precision therapies for specific TAM subpopulations. Exploring emerging areas, such as the gut microbiota‒immunometabolism axis, also represents a key frontier.

### 9.1 Current Clinical Landscape: Insights From Trials

Therapeutic strategies targeting TAM metabolism are advancing from preclinical validation to early‐phase clinical trials, representing a significant shift in cancer immunotherapy. Several metabolic modulators are now under active clinical investigation, informed by insights into TAM bioenergetic requirements. Glutamine antagonists, such as the prodrugs JHU083 and DRP‐104, disrupt immunosuppressive TAM polarization by inducing a metabolic shift toward glycolysis while simultaneously impairing oxidative phosphorylation. This metabolic rewiring enhances phagocytic ability and promotes pro‐inflammatory signatures, effects observed in prostate and bladder cancer models [150, 180]. Beyond glutamine blockade, CD40 agonists exemplify how metabolic reprogramming drives therapeutic efficacy: preclinical data reveal that CD40 ligation activates FAO and glutaminolysis in TAMs, fueling epigenetic alterations that convert pro‐tumorigenic macrophages into potent anti‐tumor effectors. This dual metabolic‒immunologic mechanism underscores why CD40 agonists achieve stronger responses in tumors lacking neoantigens, where traditional T‐cell therapies fail. Concurrently, inhibitors targeting immune checkpoints on TAMs, including blockers of TREM2 or CSF1R, demonstrate dual mechanisms: they suppress lipid accumulation and reverse glycolytic repression. For example, the TREM2 antagonist PY314 reduces TAM subsets in platinum‐resistant ovarian cancer and synergizes with pembrolizumab to improve therapeutic efficacy [181]. Clinical trials (e.g., NCT04691375 and NCT03153410) are evaluating these agents both as monotherapies and in combination regimens [182, 183]. However, a critical gap persists in the understanding of organ‐specific metabolic adaptations; for example, TAMs in hypoxic core regions may resist therapies that are effective at the invasive front because of HIF‐1α‐driven metabolic flexibility. Future trials must stratify patients by spatial metabolic heterogeneity via advanced imaging. TLR agonists such as CpG‐ODNs constitute another frontier; CpG induces a strong Th1‐type immune response (IFN‐α/γ, TNF‐α, etc.) by activating TLR9, reshaping the inhibitory components in the TME [184, 185, 186]. Trials such as NCT03445533 are exploring intratumoral CpG delivery to remodel the myeloid microenvironment in melanoma [69, 187]. Notably, the therapeutic efficacy of CpG relies on its ability to reprogram mitochondrial metabolism in TAMs. By enhancing OXPHOS, CpG enables TAMs to overcome “don't‐eat‐me” signals displayed by tumor cells. This strategy exploits a specific metabolic vulnerability absent in tumors harboring defective TCA cycles. Novel agents targeting the lipid shuttle between TAMs and tumor cells are advancing through early development. These compounds aim to disrupt metabolic symbiosis wherein lipid‐laden TAMs sustain lipid‐dependent tumor cells. The next frontier involves “metabolic combination”: simultaneously targeting compensatory pathways (e.g., COX‐2 inhibition to block PGE_2_‐driven lipid synthesis while disrupting cholesterol export via ABCG1) to prevent TAM adaptation. Key therapeutic approaches in clinical development for disrupting TAM–tumor metabolic symbiosis are outlined in Table 3.

**TABLE 3:** Clinically advanced metabolic targeting strategies for TAM–tumor symbiosis.

Therapeutic approach	Target/mode of action		Mechanism of disruption	Clinical stage	Key challenges
Glutamine antagonists	Glutaminase (e.g., JHU083, DRP‐104)	Starves tumor cells; reprograms TAM glycolysis—enhances phagocytosis [145, 157]		Phase I/II (NCT04882371)	Systemic immune toxicity; compensatory AA metabolism
CD36 monoclonal antibodies	Fatty acid transfer (e.g., CVX‐045)	Blocks lipid uptake by tumor cells; depletes lipid‐laden TAMs [161]		Phase I (NCT04721387)	Heterogeneous CD36 expression in TAM subsets
ACOD1‐deficient CAR‐macrophages	Itaconate production	ROS overproduction via disrupted TCA cycle; enhances tumoricidal activity [91]		Phase I (NCT04660929)	Limited tumor infiltration; manufacturing scalability
FAO inhibitors (neoadjuvant)	CPT1A (e.g., perhexiline)	Depletes lipid‐loaded TAMs; creates “immune‐permissive window” for ICB [160]		Phase II (melanoma)	Cardiac toxicity; requires precise sequencing
TREM2 antagonists	Lipid sensing (e.g., PY314)	Inhibits PPARγ‐driven FAO; reverses TAM immunosuppression [181, 182]		Phase Ib (NCT04691375)	On‐target microglial toxicity in CNS tumors
Nanoparticles: pH sensitive	DGAT1/2 in TAMs (e.g., DGATi‐liposomes)	Disrupts lipid droplet formation; reprograms TAMs in acidic TME [170]		Preclinical → IND‐enabling	Biodistribution to fibrotic niches; payload leakage
COX‐2 inhibitors + anti‐PD‐1	PGE_2_‒EP4 axis (e.g., celecoxib)	Reduces PD‐L1 on TAMs; reverses CD8⁺ T‐cell exhaustion [158, 164]		Phase III (NCT03894891)	Cardiovascular risks; variable PGE_2_ levels in TME
LXR agonists (e.g., RGX‐104)	ABCA1/G1‐mediated cholesterol efflux	Repolarizes TAMs via cholesterol depletion → enhances immunogenicity [188]		Phase II (NCT02922764)	Hypertriglyceridemia; hepatic steatosis
scFv‐CD206 siRNA carriers	Lipid shuttle genes (e.g., FABP5, PLIN2)	Silences lipid storage genes in TAMs; spares systemic macrophages [64, 79]		Preclinical	Endosomal escape efficiency; target saturation
CCR2 inhibitors (neoadjuvant)	Monocyte recruitment (e.g., PF‐04136309)	Depletes TAM precursors; suppresses metastasis‐initiating niches [162, 163]		Phase I (pancreatic CA)	Compensatory CXCR2‐mediated recruitment

*Data sources*: Clinical registration website.

### 9.2 Biomarker Development for Precision Targeting

Accurate biomarkers are essential for effective patient selection and therapeutic monitoring in TAM‐targeted clinical trials. Current efforts concentrate on multidimensional profiling of metabolic enzymes, circulating metabolites, and spatial imaging signatures. Transcriptomic analyses revealed that specific TAM subsets, particularly lipid‐associated macrophages (LAMs) expressing TREM2 and FASN, are correlated with poor prognosis in patients with intrahepatic cholangiocarcinoma and triple‐negative breast cancer, suggesting their potential as tissue‐based biomarkers [154, 189, 190, 191, 192]. Recent advancements in single‐cell metabolomics have facilitated the identification of functional states in immune cells beyond mere surface marker expression. Notably, the integration of ARG‐1 enzymatic activity with histone lactylation levels allows for the differentiation of immunosuppressive TAMs from their pro‐inflammatory counterparts. Additionally, the ratio of itaconate to succinate within TAMs has emerged as a predictor of glioma responsiveness to IDH1 inhibitors. Metabolomic profiling of patient serum has identified lactate and succinate as surrogate indicators of TAM activity, with elevated levels correlating with immunosuppression and resistance to immune checkpoint inhibitors. However, serum biomarkers inherently lack spatial resolution. This limitation is addressed by hyperpolarized ^13^C‐pyruvate MRI, which maps regional variations in TAM glycolysis. This technique identifies distinct “metabolic hotspots,” thereby pinpointing locations where therapeutic strategies, such as LDHA inhibition, would likely be most effective. Advanced imaging techniques, such as hyperpolarized MRI, enable non‐invasive mapping of tumor regions by mapping the conversion of ^13^C‐pyruvate [106]. Liquid biopsies analyzing tumor‐derived exosomes offer further insights into TAM reprogramming. For example, exosomal miR‐505‐3p upregulates MELK expression and plays a role in TAM polarization and T‐cell recruitment in hepatocellular carcinoma [193]. The standardization of biomarkers remains challenging, however, owing to significant interpatient variability influenced by microbiota‐derived metabolites (e.g., tryptophan catabolites) that modulate TAM function. Emerging single‐cell metabolomic platforms aim to address this by correlating in situ enzyme activity (e.g., in ARG1⁺/IDO1⁺ TAMs) with epigenetic states [194, 195]. Validation cohorts within ongoing trials (NCT04471415 and NCT06027086) will determine whether combinatorial biomarker panels—integrating factors such as ACOD1 expression, histone lactylation, and kynurenine/tryptophan ratios, which can reliably predict responses to therapies such as glutaminase inhibitors or CD40 agonists. Assessing the spatial distribution and density of lipid droplets within TAMs is a promising approach. This assessment could utilize specialized PET tracers or AI‐driven histopathology. Its potential lies in identifying patients most likely to benefit from therapies targeting TAM lipid metabolism. The clinically validated biomarkers currently under investigation are summarized in Table 4.

**TABLE 4:** Clinically validated biomarkers for TAM‐targeted therapies.

Biomarker	Detection method	Therapeutic context	Original concept linkage
CD163⁺/FABP5⁺ TAM spatial density	Multiplex IHC (AQUA)	CD36/FAO inhibitors	Lipid droplet accumulation in TAMs [52]
Plasma sCD206	Electrochemiluminescence	CSF1R antagonists	M2‐like TAM density predicts resistance [117]
TAM‐derived miR‐21‐5p	ddPCR of serum EVs	Statin + anti‐PD‐1	Exosomes transport lipids [78]
^18^F‐FTHA SUVmax (PLIN2)	PET/CT	Etomoxir‐based therapy	FABP5 inhibition sensitizes [64]
HDL‐associated SAA1	LC‒MS/MS	LXR agonists (RGX‐104)	Cholesterol efflux modulation [188]
PGE_2_ in tumor interstitial fluid	Microdialysis + ELISA	COX‐2 inhibitors	AA/PGE_2_ signaling drives polarization [90]
Lipid droplet index	Intraoperative Raman spectroscopy	Nanocarrier delivery monitoring	Lipid droplets as metabolic checkpoints [52]
Mitochondrial DNA copies in TAMs	scRNA‐seq qPCR	OXPHOS inhibitors (IACS‐010759)	β‐Oxidation fuels TAM energy [91]
CD36⁺ circulating monocytes	Flow cytometry	CVX‐045 (anti‐CD36)	CD36 mediates lipid uptake [61]
Arginase‐1 activity	^13^C‐citrulline metabolic imaging	Arginase inhibitors (CB‐1158)	Arginine metabolism in immunosuppression [65]
LXR activation signature	Nanostring nCounter	Combinatorial LXR/PD‐1 therapy	LXR/ABCA1 reprograms TAMs [188]

### 9.3 Emerging Frontiers

The future of TAM metabolic modulation lies at the intersection of synthetic biology, nanotechnology, and systems immunology. Spatial multi‐omics and advanced modeling techniques are transforming our understanding of metabolic heterogeneity within TAMs. Spatial metabolomics, which achieves single‐cell resolution, reveals dynamic metabolic gradients (e.g., itaconate vs. α‐ketoglutarate) within the TME. These gradients define metabolic niches that drive TAM functional polarization toward immunosuppressive phenotypes. Integrating this technology with organoid and 3D bioprinted models allows simulation of in vivo metabolic crosstalk networks, accelerating therapeutic target discovery. Adoptive cell therapies are evolving beyond CAR‐T cells to include metabolically engineered CAR‐Ms. Preclinical studies have demonstrated that ACOD1‐deficient CAR‐Ms exhibit enhanced tumoricidal activity through ROS overproduction, whereas LDHA knockout augments their anti‐tumor polarization [91]. The optimization of engineered cell therapies, such as CAR‐M, requires metabolic engineering strategies. For example, CRISPR‐Cas9‐mediated knockout of ACOD1 or overexpression of kynureninase can block key microenvironmental inhibitory signals. By combining this approach with spatially resolved transcriptomics to identify and localize specific TAM subpopulations, we can design CAR‐M‐cell therapies to precisely target metabolic pathways. For example, targeting lipid metabolism in subsets such as TREM2⁺ LAMs enhances CAR‐M infiltration into solid tumors. Ongoing trials (e.g., NCT04660929) are evaluating first‐generation CAR‐Ms [173]. Future iterations may co‐express metabolite‐clearing enzymes such as kynureninase to counteract the immunosuppressive microenvironment. Nanomedicine offers promising precision delivery solutions. For example, pH‐sensitive nanoparticles loaded with DGAT inhibitors can specifically deplete lipid droplets in TAMs within the acidic TME while sparing systemic tissues. Similarly, SR‐targeted micelles (e.g., directed at CD206) deliver agents such as metformin to disrupt mitochondrial complex I in prostate cancer TAMs, reversing PD‐L1 upregulation. AI is transforming the design of nanocarriers. Deep learning algorithms integrate multi‐omics data, such as combined metabolomic and epigenomic analyses, to predict metabolic vulnerabilities in TAMs and optimize the spatiotemporal release kinetics of drug delivery systems. For example, AI‐generated topological nanoparticle structures enhance penetration into fibrotic tumors, enabling targeted inhibition of key enzymes (e.g., DGAT1/2) in lipid shuttle pathways. In addition to therapeutics, AI‐driven models that integrate multi‐omics datasets are used to identify context‐dependent metabolic vulnerabilities. For example, hypoxic tumors that exhibit HIF‐1α‐driven TAM glycolysis may respond optimally to MCT4 inhibitors. Novel metabolic/epigenetic targets offer new axes for combination therapy. Histone lactylation inhibitors block IL‐10 transcription, whereas small‐molecule antagonists of the ACOD1‒itaconate axis reverse CD8⁺ T‐cell exhaustion. Rational combination with immune checkpoint inhibitors synergistically reprograms the immune microenvironment. The gut–tumor axis represents another frontier: modulating ureolytic bacteria reduces polyamine synthesis in colorectal TAMs, a strategy under investigation in microbiome engineering trials. Engineered bacteria shows promise as vehicles for targeted epigenetic editing. Probiotics modified via synthetic biology techniques can deliver HDAC inhibitors directly to CRC niches. This approach effectively downregulates ARG‐1 expression in TAMs. The efficacy of such strategies can be dynamically assessed via spatial multi‐omics data to inform computational models of microbe‒host metabolic interactions. Finally, spatial metabolomics platforms that map metabolite gradients (e.g., itaconate vs. α‐ketoglutarate) at single‐cell resolution will uncover novel targets. This could transition the field from simply blocking TAM functions to actively reprogramming them to promote anti‐tumor immunity. Engineering macrophages to disrupt pathological lipid transfer represents a promising frontier strategy. Specific methods include knocking down gene expression in CAR‐M cells or delivering blocking biologics via TAM‐tropic nanoparticles. This approach aims to dismantle the metastatic cascade fueled by TAMs.

## 10 Discussion

The dynamic reciprocity between tumor cells and TAMs extends beyond established immune evasion mechanisms [196, 197]. Metabolic reprogramming has emerged as a fundamental driver of pathological polarization within the TME. This co‐evolutionary relationship manifests as bidirectional metabolic crosstalk. Tumor cells and TAMs reciprocally alter each other's functional states through secreted metabolites, extracellular vesicles, and competition for nutrients. Crucially, alterations in lipid metabolism act as master regulators of this interaction. Under metabolic stress, tumor cells accumulate lipid droplets and release exosomes enriched with fatty acids or PGE_2_. These engage receptors such as CD36 or PPARγ on TAMs, enforcing immunosuppressive polarization. In parallel, TAMs reciprocate by supplying tumor cells with critical metabolic substrates such as free fatty acids, cholesterol, and succinate. These compounds fuel invasion, stemness, and therapy resistance. This metabolic symbiosis creates a self‐amplifying circuit: polarized tumor cells generate signals that lock TAMs into pro‐tumoral states, whereas M2‐like TAMs further increase tumor aggressiveness through metabolite‐driven epigenetic and transcriptional changes [198, 199].

The spatial organization of the metabolic microenvironment within tumors further reinforces this interdependence. Hypoxic regions stabilize HIF‐1α. This drives tumor cell lactate overproduction and enhances its uptake by TAMs via MCT1 and MCT4 [200, 201, 202]. Intracellular lactate then induces histone lactylation in TAMs. This epigenetic modification represses anti‐tumor genes while activating IL‐6/STAT3 signaling [203]. Furthermore, TAM‐derived itaconate, generated when ACOD1 diverts the TCA cycle, promotes cancer cell mutagenesis through ROS‐dependent DNA damage [204]. Such compartmentalized metabolite exchange demonstrates how metabolic byproducts directly modulate transcriptional programs across cellular boundaries, going beyond mere energy provision. Furthermore, amino acid competition adds complexity to this landscape. Tumor cells overexpressing glutamine‐fructose‐6‐phosphate transaminase 2 (GFPT2) deplete extracellular glutamine, impairing mitochondrial fission and the phagocytic capacity of TAMs [205]. Conversely, TAMs expressing ARG‐1 or indoleamine 2,3‐dioxygenase (IDO) deplete arginine and tryptophan, starving cytotoxic T cells while producing immunosuppressive kynurenine [206].

Targeting this metabolic axis offers compelling intervention strategies, yet its complexity necessitates nuanced approaches. Small‐molecule inhibitors that disrupt key nodes, such as glutamine antagonists (JHU083, DRP‐104) or TCA cycle modulators, demonstrate dual efficacy. They starve tumor cells while simultaneously reprogramming TAM metabolism toward pro‐inflammatory states. For example, JHU083 antagonism enhances TAM glycolysis and phagocytosis while increasing the antigen presentation machinery. Similarly, COX‐2 inhibitors such as celecoxib reduce PGE_2_‐mediated immunosuppression and downregulate PD‐L1 expression on TAMs, synergizing with immune checkpoint blockade. Nanotechnology platforms improve specificity by leveraging SRs, such as CD206 or MARCO, or exploiting the acidic pH to deliver agents such as DGAT inhibitors or metformin specifically to TAMs [107]. This depletes lipid droplets without causing systemic toxicity. Emerging adoptive cell therapies, such as CAR‐Ms engineered to lack ACOD1, exploit metabolic rewiring to increase ROS production and tumoricidal activity.

However, clinical translation faces significant hurdles. Metabolic heterogeneity across TAM subsets, revealed by techniques such as single‐cell lipidomics and spatial transcriptomics, complicates broad targeting. For example, lipid‐laden TAMs in necrotic zones exhibit distinct cardiolipin saturation and transporter expression, such as upregulated MDR1, compared with perivascular subsets, influencing drug retention. Moreover, therapeutic pressure risks triggering paradoxical adaptations. Glutamine restriction may upregulate alternative amino acid catabolism pathways, whereas TAM depletion via CSF1R inhibitors can inadvertently increase PD‐L1 expression in the remaining TAM population. The plasticity of polarization states further challenges treatment durability. Epigenetic memory mechanisms, including lactylation‐stabilized PPARγ promoters, enable TAMs to rapidly revert to immunosuppressive phenotypes after therapy cessation. Consequently, rational combination strategies and sequencing are essential. Preclinical data suggest that neoadjuvant FAO inhibitors deplete lipid‐loaded TAMs before initiating checkpoint blockade, potentially creating an “immune‐permissive window.” Timing therapeutics to align with intrinsic metabolic rhythms, such as those regulated by REV‐ERα in lipid uptake, is another promising strategy. Disruption of the lipid transfer pathway has significant potential. However, its effectiveness requires approaches that address metabolic heterogeneity among TAMs. To maximize efficacy, strategies could include nanoparticle delivery systems targeting specific TAM subsets rich in lipid droplets. Alternatively, combining lipid transfer blockade with agents that inhibit lipid utilization by tumor cells, such as FAO inhibitors, is another promising approach.

Despite mechanistic advances, persistent knowledge gaps hinder clinical progress. First, the initial metabolic triggers driving TAM polarization remain elusive. While tumor‐derived lactate or lipids are implicated, their spatiotemporal dynamics during early neoplasia, when polarization might be most therapeutically reversible. In addition, robust in vivo validation is lacking. Second, limitations in metabolite detection impede the establishment of causal relationships. Current technologies cannot resolve real‐time metabolic flux between individual tumor cell‒TAM pairs, forcing reliance on bulk analyses that obscure cellular heterogeneity. Third, evolutionary adaptations undermine therapeutic durability. Metabolic inhibitors impose selection pressures favoring tumor subclones with compensatory pathways, such as FAS upregulation during cholesterol blockade or the maintenance of epigenetic “memory” of immunosuppressive programming by TAMs.

Technical barriers further complicate targeting. Drug delivery systems struggle to penetrate fibrotic tumor regions where lipid‐laden TAMs reside, whereas heterogeneity in SR expression, such as variable CD206 or MARCO levels across metastases, limits nanoparticle precision. Crucially, interspecies metabolic differences obscure human relevance. Compared with their human counterparts, murine TAMs handle lipids distinctly, and xenograft models fail to replicate the nutrient gradients characteristic of the human TME.

The profound biological importance of TAMs remains paramount, driven by their critical roles in cancer inception, advancement, and metastatic dissemination [207]. While significant progress has been made in elucidating the mechanistic interactions between TAMs and malignant cells, a comprehensive understanding of the metabolic pathways governing TAM functionality is lacking. Foundational insights derived from studies of LPS‐ or IL‐4‐induced macrophage metabolism under controlled conditions provide valuable models for controlling polarization. However, the dynamic heterogeneity and unique ecological constraints of the TME necessitate careful consideration. Importantly, research into essential metabolic pathways directly involves macrophages within diseased tissues, which are continuously exposed to the complex signaling cues characteristic of the TME. Despite ongoing challenges, the pursuit of understanding TAM metabolism has yielded significant discoveries, leading to the development of promising therapeutic strategies. Concurrently, the continuous advancement of tools for immunometabolism analysis at single‐cell resolution promises a more detailed examination of TAM metabolic complexities. This expanding knowledge base, in turn, supports innovative strategies aimed at strategically disrupting the pathological symbiosis between TAMs and cancer. Targeting metabolic symbiosis, particularly lipid transfer between TAMs and cancer cells, represents a highly promising therapeutic frontier. This potential necessitates further development of spatial drug delivery systems, exploration of combination therapies, and identification of robust biomarkers to facilitate clinical translation.

## Author Contributions

Guohao Wei and Bin Li wrote the article. Guohao Wei, Bin Li, Mengyao Lv, and Zihui Liang designed the figure and organized the table. Mengyang Huang, Lilin Ge, Jing Chen, and Chuandong Zhu contributed to the data resources. Guohao Wei, Bin Li, Mengyang Huang, Mengyao Lv, and Zihui Liang analyzed literature. Chuandong Zhu, Lilin Ge, and Jing Chen administrated the organization, implementation, supervision, and funding acquisition of the whole review. All the authors have read and approved the final manuscript.

## Ethics Statement

The authors have nothing to report.

## Conflicts of Interest

The authors declare no conflicts of interest.

## Data Availability

The authors have nothing to report.
